# Development and initial psychometric evaluation of a digital competence scale for kindergarten teachers in Western China

**DOI:** 10.1186/s40359-026-04551-0

**Published:** 2026-04-21

**Authors:** Tao Tian, Shuyu Huang, Huina Yu, Siyang Wei, Huixuan Yu

**Affiliations:** College of Teachers, Chengdu University, 2025 Chengluo Avenue, Chengdu, Sichuan 610106 China

**Keywords:** Digital competence, Kindergarten teachers, Digital literacy, Early childhood education and care, Ethnic minority areas, Scale development

## Abstract

**Purpose:**

To develop and validate a scale assessing kindergarten teachers’ digital competence in ethnic minority areas of Western China amid growing ECEC digitalisation demands, where digital infrastructure, access to resources, and professional learning opportunities may be uneven.

**Methodology:**

A total of 764 kindergarten teachers completed a 29-item instrument covering five domains: digital awareness, digital skills, digital application, digital social responsibility, and professional development. Internal consistency (Cronbach’s α), Spearman–Brown split-half reliability, corrected item–total correlations, split-sample exploratory factor analysis, confirmatory factor analysis, and known-groups comparisons were examined.

**Findings:**

The scale demonstrated excellent reliability (total α = 0.980; subscale αs = 0.895–0.969) and strong split-half reliability (total SB = 0.990; subscale SBs = 0.903–0.981). The structural analyses indicated that the theoretically specified five-factor model fit the data better than both three-factor and one-factor alternatives. Corrected item–total correlations were uniformly adequate, while the very high total reliability suggested some degree of item overlap. Known-groups comparisons also provided preliminary external validity evidence.

**Implications:**

The instrument provides a reliable, multidimensional tool for assessing ECEC teachers’ digital competence and can support diagnostic profiling and targeted professional development, particularly in under-resourced, culturally diverse, and regionally uneven early childhood contexts.

**Supplementary Information:**

The online version contains supplementary material available at 10.1186/s40359-026-04551-0.

## Introduction

Digitalisation is reshaping education systems worldwide, altering how teaching is planned, delivered, assessed, and documented. In this context, teachers are increasingly expected to make informed decisions about digital resources and tools, integrate them into pedagogy, and adapt professional practices to technology-mediated environments. As a result, “digital competence” has become a key construct for understanding educators’ capacity to navigate digitally saturated learning settings, and it is now widely recognised as more than technical proficiency alone [1].

Contemporary frameworks emphasise that teachers’ digital competence is multidimensional and includes pedagogical use of technologies, professional engagement, and ongoing development. For example, the European DigCompEdu framework conceptualises educators’ competence as a structured set of domains spanning professional practices, instructional design and delivery, assessment, learner empowerment, and facilitating learners’ own digital competence. Similarly, UNESCO’s ICT Competency Framework for Teachers highlights that teacher competence involves not only classroom-level practice but also broader responsibilities linked to policy, curriculum, ethics, and professional development. These perspectives converge in portraying digital competence as an integrated capability that combines knowledge, skills, and professional judgement in context [1–4].

In early childhood education and care (ECEC), the digital turn raises distinctive demands. Early childhood teachers must ensure that technology use is developmentally appropriate and supportive of children’s well-being, while also engaging families and safeguarding children in digital environments. Recent OECD work underscores that digitalisation places new requirements on the ECEC workforce, including the ability to use digital tools in ways that align with early years pedagogy and to respond to emerging issues such as children’s digital experiences, protection, and care in the digital age [5].

Within China, teacher digital competence has been elevated to a formal standardisation and governance agenda. In policy terms, this construct is operationalised through the Ministry of Education’s teacher digital literacy standard. The Ministry of Education’s industry standard *Digital literacy of teachers (JY/T 0646—2022)* specifies a five-dimensional framework that includes digital awareness, digital skills, digital application, digital social responsibility, and professional development, providing an explicit reference for teacher training and evaluation. This policy context underscores the importance of robust measurement and evidence that can support implementation and improvement, particularly in educational settings facing uneven resources and diverse contextual needs.

## Literature review

### Conceptualising teachers’ digital competence

Across international frameworks, teachers’ digital competence is generally defined as an integrated capability that combines knowledge, skills, and professional judgement for using digital technologies to support teaching and learning [2, 6]. DigCompEdu positions educators’ competence as a structured set of domains covering professional engagement, digital resources, teaching and learning, assessment, learner empowerment, and supporting learners’ digital competence. Importantly, DigCompEdu treats competence as pedagogically grounded rather than purely technical, highlighting educators’ decision-making and instructional design in digital contexts [1]. In a complementary way, UNESCO’s ICT Competency Framework for Teachers (ICT-CFT) emphasises competence as a policy- and system-facing construct: it links classroom technology use to curriculum and assessment, school organisation, and teacher professional learning, with attention to equity and responsible use [4].

China’s teacher digital literacy standard (JY/T 0646—2022) provides a nationally codified model that is closely aligned with the “multi-domain” view found in these global frameworks. It specifies five dimensions—digital awareness, digital skills, digital application, digital social responsibility, and professional development— offering a policy-relevant structure for training and evaluation. This alignment across international and national frameworks suggests that teacher digital competence is best treated as a multidimensional construct rather than a single undifferentiated trait.

### Digital competence in early childhood education and care

Digital competence in early childhood education and care (ECEC) is often discussed as a special case because the goals and constraints of early years pedagogy differ from those in later schooling. The OECD’s synthesis on professional development for digital competencies in ECEC highlights that digitalisation introduces new demands for educators to support children’s learning and well-being in the digital age, and that competence requirements can vary by role (e.g., classroom educators, leaders, specialist staff) and by local governance and infrastructure [5]. Related OECD work further underscores that young children’s digital experiences bring both opportunities and risks, implying that ECEC settings must balance pedagogical enrichment with safeguarding and developmentally appropriate practice [7].

Empirical reviews focusing on China echo these points. A systematic review of early childhood teachers’ readiness for digital transformation in Mainland China concluded that readiness remains uneven and that teacher education and professional development are central levers for improving competence and confidence in technology integration [8]. Together, these syntheses indicate that ECEC teachers’ digital competence should be examined in frameworks that capture pedagogy-specific demands (e.g., documentation of child development, family communication) as well as contextual constraints (e.g., resource availability, training access).

Methodological precedents from adjacent ECEC fields are also informative for the present study. For example, Losada-Puente et al., [9] developed and validated a multidimensional instrument of early childhood teachers’ professional development in physical education through literature analysis, expert judgment, pilot testing, exploratory factor analysis, and confirmatory factor analysis. Although this work addressed physical education rather than digital competence, it is methodologically relevant because it demonstrates how a domain-specific teacher-competence instrument can be constructed and psychometrically evaluated within an early childhood context.

### Digital social responsibility and “digital citizenship” in teachers’ work

A recurring theme across frameworks is that competent digital practice involves ethical and responsible use—especially in relation to privacy, data protection, and safe participation in digital environments. UNESCO’s ICT-CFT explicitly links digital competence to broader responsibilities such as equitable participation and responsible technology use [4]. In the research literature, responsibility-related aspects are often framed as teachers’ digital citizenship, typically involving norms, behaviours, and dispositions for safe, ethical, and constructive engagement online [10]. For example, evidence from teacher samples suggests that digital citizenship levels vary systematically with personal and contextual factors, implying that “responsible” dimensions are not automatically ensured by technical skills alone [11].

In ECEC, responsibility may be particularly salient because teachers routinely handle sensitive child-related information (e.g., developmental records, family contact information, learning documentation) and must consider children’s safety and well-being in digital environments. OECD analyses of the early childhood digital landscape similarly emphasise that managing risks and safeguarding children are core concerns in digitally mediated early years settings [5]. This implies that an ECEC-oriented model of teacher digital competence should treat responsible practice as a central domain rather than a peripheral add-on.

### Measurement and professional reflection tools

In parallel with conceptual development, measurement efforts have expanded through scale development and validation studies, reflecting demand for instruments that can support both research and teacher development. For instance, recent work has developed comprehensive teacher digital competence scales and provided evidence for reliability and factorial validity across teacher populations (Aydin & Yildirim, 2024) [12]. Beyond psychometric instruments, structured self-reflection resources such as the SELFIEforTEACHERS toolkit are increasingly used to help educators review their digital practices and guide professional learning and improvement processes [13]. Evidence from psychometric analyses of the related SELFIE self-reflection tool also supports the value of such structured reflection instruments for monitoring and strengthening digital capacity [14].

However, the measurement literature also suggests a practical challenge: many instruments are either (a) broadly targeted at teachers across educational levels, or (b) focused primarily on technology integration skills without an equally strong representation of responsibility-related practices. This highlights the value of instruments that are explicitly aligned with an authoritative standard (e.g., JY/T 0646—2022) and that are tailored to ECEC work realities.

Recent evidence from adjacent early childhood domains also suggests that measuring teachers’ professional competence is practically meaningful because teacher-level competence may relate to child-level developmental outcomes. In physical education, Honrubia Montesinos et al., [15] reported that components of early childhood teachers’ professional development predicted children’s fundamental movement skills. Although digital competence is a different domain, this line of evidence strengthens the practical rationale for assessing kindergarten teachers’ professional competence in ways that can inform targeted support and improvement.

### The current study

Building on China’s teacher digital literacy standard (JY/T 0646—2022) and convergent international frameworks, the present study evaluates a 29-item measure of kindergarten teachers’ digital competence in ethnic minority areas of Western China [1, 4]. We conceptualise digital competence as a multidimensional construct comprising digital awareness, digital skills, digital application, digital social responsibility, and professional development. The study aims to provide psychometric evidence for this instrument’s reliability and, structural validity, and preliminary external validity in an ECEC context where digitalisation demands and professional learning opportunities may be uneven [5, 8].

Accordingly, we address three research questions: (1) Does the scale demonstrate satisfactory internal consistency reliability for the total score and each subscale? (2) Is there structural evidence supporting the intended dimensional organisation of the instrument based on a split-sample exploratory and confirmatory approach? (3) Does the theoretically specified five-factor model fit the data better than three-factor and one-factor alternatives? (4) Do scale scores differ in theoretically meaningful ways across teacher background groups? Establishing these properties would support the instrument’s use for descriptive assessment and for informing targeted professional development aligned with national requirements [5].

## Method

### Participants and procedure

Kindergarten teachers working in ethnic minority areas of Western China participated in this study. The analytic sample comprised 764 teachers with complete responses on all scale items. Participants were drawn primarily from Sichuan (*n* = 387, 50.7%) and Tibet (*n* = 339, 44.4%), with smaller numbers from Guangxi (*n* = 27, 3.5%), Guizhou (*n* = 4, 0.5%), and other provinces (total *n* = 7, 0.9%). The sample included 111 men (14.5%) and 653 women (85.5%). Age was grouped as ≤ 25 years (*n* = 199, 26.0%), 26–35 years (*n* = 437, 57.2%), 36–45 years (*n* = 71, 9.3%), and ≥ 46 years (*n* = 57, 7.5%). Teaching experience was ≤ 3 years (*n* = 217, 28.4%), 3–5 years (*n* = 174, 22.8%), 5–10 years (*n* = 228, 29.8%), 10–15 years (*n* = 51, 6.7%), and > 15 years (*n* = 94, 12.3%). Additional background characteristics are reported in Table 1. Because complete invitation-denominator information was not retained, a response rate could not be calculated; accordingly, the sample should be interpreted as regionally informative rather than population-representative.

**Table 1:** Participant Characteristics (*N* = 764)

Variable	Category	*n*	%
Sex	Male	111	14.5
	Female	653	85.5
Age	≤ 25 years	199	26.0
	26–35 years	437	57.2
	36–45 years	71	9.3
	≥ 46 years	57	7.5
Teaching experience	≤ 3 years	217	28.4
	3–5 years	174	22.8
	5–10 years	228	29.8
	10–15 years	51	6.7
	> 15 years	94	12.3
Academic qualification	High school or below	26	3.4
	Junior college	391	51.2
	Bachelor’s degree	346	45.3
	Master’s degree or above	1	0.1
Location	Urban	244	31.9
	Township	360	47.1
	Rural	160	20.9
Kindergarten type	Public	707	92.5
	Inclusive private	48	6.3
	For-profit private	9	1.2
Job title	Junior	250	32.7
	Intermediate	115	15.1
	Senior	32	4.2
	Unrated	367	48.0
Professional background	Teacher education	590	77.2
	Non-teacher education	174	22.8

### Measure

Digital competence was assessed using 29 items (q1–q29) rated on a 5-point Likert scale (1 = strongly disagree, 5 = strongly agree The questionnaire was developed drawing primarily on China’s teacher digital literacy standard (JY/T 0646—2022) and on instruments reported by Chen and Min [16] and Yu and Zhang [17]. In addition, the present measurement approach was informed by adjacent ECEC instrument-validation work showing the value of multidimensional, domain-specific teacher-competence measures and staged psychometric validation procedures (e.g., Losada-Puente et al., [9]. Higher scores indicate stronger digital competence. For descriptive and correlational analyses, items were aggregated into five theoretically specified domains: Digital Awareness (q1–q5), Digital Skills (q6–q8), Digital Application (q9–q18), Digital Social Responsibility (q19–q24), and Professional Development (q25–q29). In addition to these domain scores, we examined whether the item set could be summarized parsimoniously as three broader clusters—Awareness (q1–q5), Practice (q6–q18; integrating skills and application), and Norms & Development (q19–q29; integrating social responsibility and professional development)— as an alternative higher-level summary of the data. However, the primary measurement structure evaluated in this study remained the five-domain framework aligned with JY/T 0646—2022. Because formal expert-panel ratings, content-validity indices, and a separate pilot dataset were not retained, the present study is best interpreted as a psychometric evaluation of a theory- and policy-informed item pool.

### Data analysis

Analyses were conducted on the item matrix and subscale sum scores. We first computed descriptive statistics and Pearson correlations among the five domain scores [18]. Internal consistency was then evaluated using Cronbach’s α and Spearman–Brown split-half reliability (odd–even split). To examine item discrimination and possible redundancy, we additionally calculated corrected item–total correlations and α-if-item-deleted values for the full scale. The sample was randomly split into two halves of equal size (*n* = 382 each). In the calibration subsample, the suitability of the item matrix for factor analysis was assessed using the Kaiser–Meyer–Olkin (KMO) index [19] and Bartlett’s test of sphericity, followed by exploratory factor analysis. In the validation subsample, confirmatory factor analyses were conducted to compare one-factor, three-factor, and five-factor measurement models. Finally, to extend validity evidence beyond internal consistency and internal structure, we conducted known-groups comparisons using academic qualification, teaching experience, and professional background.

## Results

### Descriptive statistics and correlations

Table 2 reports means, standard deviations, and correlations among the five domain scores (sum scores). Teachers reported relatively high levels across domains, and all domains were strongly and positively correlated, indicating a coherent digital competence profile in which higher scores in one domain tended to co-occur with higher scores in the others.

**Table 2:** Means, standard deviations, and correlations among study variables (*N* = 764)

Variable	M	SD	1	2	3	4
1. Digital awareness (q1–q5)	21.760	2.880	—			
2. Digital skills (q6–q8)	12.530	2.000	0.760***	—		
3. Digital application (q9–q18)	41.520	6.430	0.710***	0.850***	—	
4. Digital social responsibility (q19–q24)	25.840	3.430	0.700***	0.660***	0.730***	—
5. Professional development (q25–q29)	21.220	3.010	0.700***	0.730***	0.790***	0.840***

Note. * p  < .05, **  < .01, *** p < .001

### Reliability

Internal consistency was excellent for all five domains and for the full scale. Split-half coefficients were computed using an odd–even item split with Spearman–Brown correction. Specifically, the Digital Awareness domain demonstrated high internal consistency (α = 0.924) and strong split-half reliability (Spearman–Brown = 0.951). Reliability was similarly strong for Digital Skills (α = 0.895, Spearman–Brown = 0.903) and was particularly high for Digital Application (α = 0.969, Spearman–Brown = 0.981). The domains reflecting responsible practice and developmental orientation also showed robust consistency: Digital Social Responsibility yielded α = 0.938 with Spearman–Brown reliability of 0.951, and Professional Development yielded α = 0.946 with Spearman–Brown reliability of 0.956. At the level of the full instrument, the total score demonstrated excellent reliability (α = 0.980, Spearman–Brown = 0.990), supporting the use of both domain scores and an overall digital competence score for research and applied interpretation. At the same time, because coefficients of this magnitude may also indicate item overlap, we further examined corrected item–total correlations and α-if-item-deleted values. Corrected item–total correlations ranged from 0.636 to 0.847, and α-if-item-deleted ranged from 0.978 to 0.979, indicating that all items contributed meaningfully but that some redundancy may be present.

### Suitability for component extraction

In the calibration subsample, the correlation matrix was highly suitable for factor analysis: KMO = 0.969 and Bartlett’s test of sphericity was significant, χ²(406) = 13642.63, *p* < .001. Exploratory factor analysis broadly supported the intended multidimensional organisation of the instrument.

### Structural model comparison

In the validation subsample, confirmatory factor analysis was used to compare one-factor, three-factor, and five-factor models. The theoretically specified five-factor model fit the data better than both the three-factor and one-factor alternatives: χ²(367) = 1400.12, CFI = 0.915, TLI = 0.906, RMSEA = 0.086. By comparison, the three-factor model showed poorer fit, χ²(374) = 1872.42, CFI = 0.877, TLI = 0.866, RMSEA = 0.103, and the one-factor model performed worst, χ²(377) = 3603.29, CFI = 0.735, TLI = 0.714, RMSEA = 0.150. Standardized factor loadings in the five-factor model ranged from 0.728 to 0.909. These findings indicate that the scale is best interpreted as assessing five correlated dimensions, whereas the three-cluster summary is better understood as a higher-level condensation of overlap among domains rather than the primary measurement structure (see Table 3).

**Table 3:** Fit indices for competing measurement models in the validation subsample (*n* = 382)

Model	χ²	df	CFI	TLI	RMSEA
One-factor	3603.29	377	0.735	0.714	0.150
Three-factor	1872.42	374	0.877	0.866	0.103
Five-factor	1400.12	367	0.915	0.906	0.086

*Note. *The five-factor model corresponds to the theoretically specified structure of Digital Awareness, Digital Skills, Digital Application, Digital Social Responsibility, and Professional Development

### Known-groups validity

To extend validity evidence beyond internal consistency and internal structure, we examined whether scores differed across theoretically relevant teacher background groups. Teachers with a bachelor’s degree or above scored higher than those with junior college education or below on the total scale, t = 2.33, *p* = .020, d = 0.17, and also on Digital Awareness, t = 2.63, *p* = .009, d = 0.19, Digital Social Responsibility, t = 3.72, *p* < .001, d = 0.27, and Professional Development, t = 2.34, *p* = .020, d = 0.17. Teachers with ≤ 5 years of experience scored higher on Digital Skills, t = 2.81, *p* = .005, d = 0.20, and Digital Application, t = 1.97, *p* = .049, d = 0.14, than those with more than 5 years of experience. Teachers with a teacher-education background scored higher than those with a non-teacher-education background on Digital Awareness, t = 2.80, *p* = .006, d = 0.23, and Professional Development, t = 2.33, *p* = .021, d = 0.20. Although these effects were small, they were directionally interpretable and therefore provide preliminary known-groups validity evidence.

### Common method considerations

Because all constructs were measured via a single self-report survey, common method variance cannot be excluded. An unrotated first-factor diagnostic indicated that the first factor accounted for 63.75% of the total variance. This diagnostic is descriptive and does not distinguish substantive general factors from method effects; therefore, inferences should be interpreted with appropriate caution and should not be taken as evidence that method bias is absent; future research may incorporate multi-source indicators or temporally separated measurement designs. Accordingly, this analysis should be treated as limited evidence only, rather than as a strong test ruling out common method bias.

## Discussion

The present study evaluated a 29-item scale designed to assess kindergarten teachers’ digital competence in ethnic minority areas of Western China, aligned with China’s teacher digital literacy standard (JY/T 0646—2022) and consistent with international competency frameworks [1, 4]. Overall, the findings provide encouraging psychometric support for the scale [20, 21]. Internal consistency was excellent for the total score and robust across all five theoretically specified subscales, indicating that the items within each domain cohere well and that the full item set yields highly reliable composite scores [22]. This reliability profile is consistent with the view that teacher digital competence is an integrated capability encompassing awareness, practical enactment, responsible conduct, and sustained professional growth [1, 4]. At the same time, the very high total reliability suggests that some item overlap may be present and should be considered in future refinement work.

Beyond reliability, the structural analyses provide stronger evidence for the instrument’s dimensional organisation. The five-factor model fit the data better than both the three-factor and one-factor alternatives, indicating that the scale is best interpreted as measuring five correlated dimensions aligned with JY/T 0646—2022. At the same time, the three-cluster summary remains useful as a higher-level description of overlap among domains rather than as the primary measurement structure.

The correlation pattern among the five subscales further supports the interpretation that the domains are closely linked rather than independent. Strong positive associations among awareness, skills, application, social responsibility, and professional development suggest that teachers’ digital competence operates as a coordinated profile: those who feel more capable and engaged in applying digital tools also tend to endorse stronger responsibility-related practices and a more proactive professional learning orientation. At the same time, the consistently high intercorrelations suggest substantial overlap among domains, underscoring the value of follow-up work using external criteria to further evaluate discriminant validity and the practical distinctiveness of the subscales. This finding echoes the competency-framework assumption that responsible use and continuous development are not optional add-ons but are integral to competent practice—especially in contexts where teachers handle sensitive child data, engage families digitally, and face emergent online risks [4, 7]. This pattern is also consistent with the idea that practice, responsibility, and development are naturally intertwined in teachers’ professional work.

Taken together, the results suggest that the five subscales provide the most defensible primary interpretation of the instrument. A total score may still be useful for broad descriptive purposes, but the present findings support the strongest interpretation at the level of five correlated domains rather than a compressed three-component structure.

### Implications

From an applied perspective, the instrument offers a practical tool for supporting the implementation of China’s teacher digital literacy standard in early childhood settings, particularly in ethnic minority areas of Western China where professional learning opportunities and digital infrastructure may be uneven [5]. Education administrators and teacher educators can use the domain scores to identify priority needs—for example, distinguishing teachers who are motivated and value-aligned (Awareness) but require support in classroom enactment (Practice), or those who use technology frequently but need guidance on privacy, data protection, and risk governance (Norms & Development). This applied orientation is also consistent with evidence from adjacent early childhood domains showing that teachers’ professional development may be associated with child-level outcomes; for example, Honrubia Montesinos et al., [15] found that teachers’ professional development in physical education predicted children’s fundamental movement skills. Although the domain differs from digital competence, both lines of work suggest that assessing teacher competence can provide a meaningful basis for targeted professional support. In addition, the integrated structure suggests that professional development may be most effective when it combines technical-pedagogical training with explicit content on responsible practice and sustained professional learning pathways, reflecting the comprehensive orientation advocated by international frameworks [1, 4].

### Limitations and future directions

Several limitations warrant consideration. First, the study relied on cross-sectional self-report data collected through a single survey, which may inflate associations among domains and cannot fully disentangle substantive general competence from shared method variance [23]. Future research should incorporate multi-source indicators (e.g., peer/leader ratings), behavioural evidence (e.g., digital artefacts, platform logs), or temporally separated measurement to strengthen validity inferences. Second, although the study now includes exploratory and confirmatory structural analyses as well as known-groups comparisons, it should still be interpreted as a psychometric evaluation of a theory- and policy-informed item pool. Formal expert-panel ratings, content-validity indices, and a separate pilot dataset were not retained. Third, because complete invitation-denominator information was not retained, a response rate could not be calculated, and the sample should not be treated as population-representative of all kindergarten teachers in Western China. Future work should also examine whether a shorter version of the scale can preserve content coverage while reducing possible item redundancy.

## Conclusion

This study evaluated a 29-item instrument to assess kindergarten teachers’ digital competence in ethnic minority areas of Western China. The scale demonstrated excellent internal consistency for the total score and strong reliability across the five subscales (digital awareness, digital skills, digital application, digital social responsibility, and professional development). The structural analyses indicated that the five-factor model fit the data better than three-factor and one-factor alternatives, and known-groups comparisons provided preliminary external validity evidence. Overall, the instrument provides a practical, standard-aligned tool for research, diagnostic assessment, and professional development planning in early childhood settings. At the same time, further refinement and external validation remain necessary.

## Supplementary Information

Supplementary Material 1.

Supplementary Material 2.

## Data Availability

Data is provided within the manuscript or supplementary information files.
