# Supplementary material for: Development and initial psychometric evaluation of a digital competence scale for kindergarten teachers in Western China
Source: BMC Psychol. 2026 Apr 21;14:809. doi: 10.1186/s40359-026-04551-0 (PMC13227872; doi:10.1186/s40359-026-04551-0)
Supplement: Supplementary file 2 — Supplementary Material 2. [file 40359_2026_4551_MOESM2_ESM.pdf]

Research Ethics Review Certificate (2024-04)

The Research Ethics Review Committee at Teachers College, Chengdu University has completed a pre-study ethics review of the project entitled “Development and Psychometric Evaluation of a Digital Competence Scale for Kindergarten Teachers in Western China.”

The study plans to recruit kindergarten teachers working in ethnic minority areas of Western China and, using random sampling, to administer a non-invasive questionnaire survey in public and private kindergartens across four provincial-level regions in China (Sichuan, Tibet, Guangxi, and Guizhou). The target participants are adult professionals, and the study does not involve clinical interventions or biological specimen collection.

Following review, the Committee confirms that the study protocol complies with the ethics regulations of Chengdu University and applicable laws and regulations governing research involving human participants, and approves the conduct of the research as described in the approved protocol. The ethics approval process included approvals dated from 10 Jan 2024 to 10 Feb 2024**,** with relevant documentation filed in accordance with institutional requirements.

The Committee notes that the protocol includes appropriate protections for participants, including voluntary participation, the right to withdraw at any time without penalty or impact on employment, confidentiality and secure handling of personal information, data minimisation, and de-identification/anonymisation procedures as appropriate; the Committee has also reviewed the proposed informed consent and data management procedures as part of the ethics application. All research procedures must be conducted in accordance with applicable ethical principles and guidelines for research involving human participants, including the principles reflected in the Declaration of Helsinki, and relevant institutional regulations. Any substantial amendments to the protocol, recruitment procedures, study sites/regions, or timelines must be submitted to the Committee for review and approval prior to implementation.

Professor Wang, Director of the Research Ethics Review Committee at Teachers College, Chengdu University.

Jan 10, 2024.
